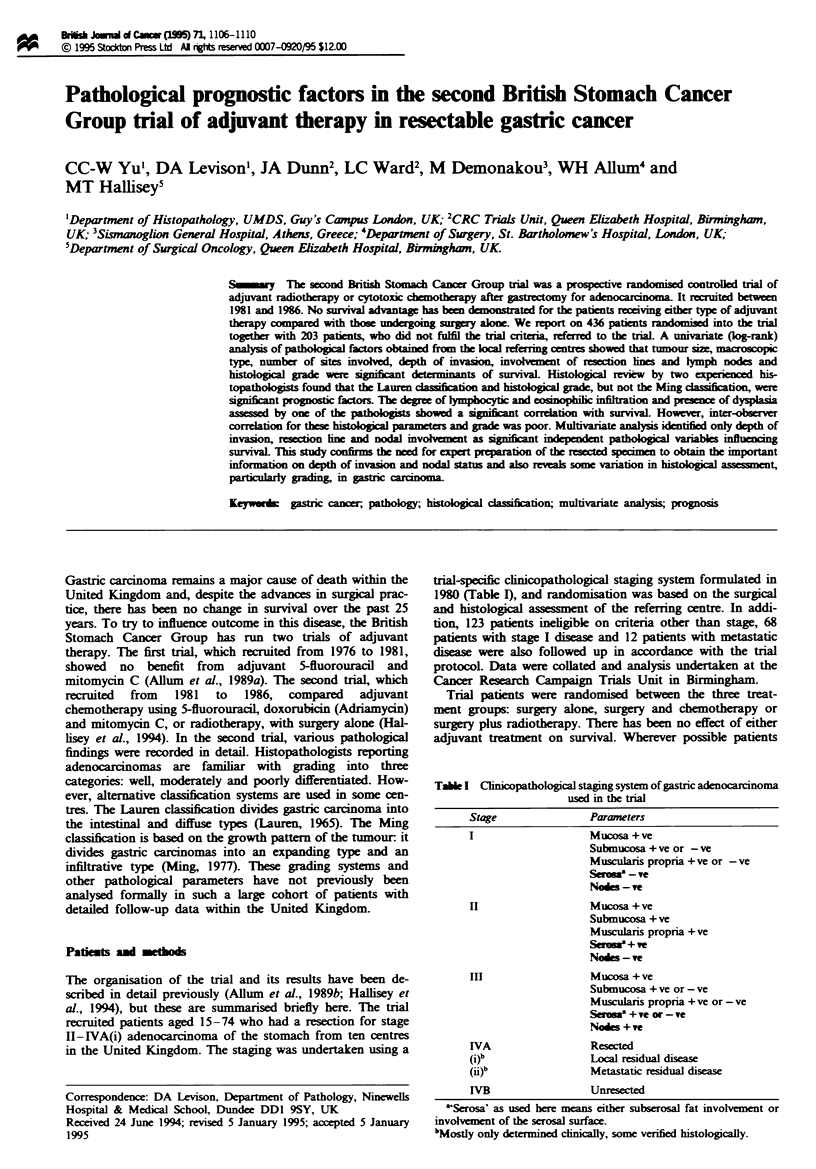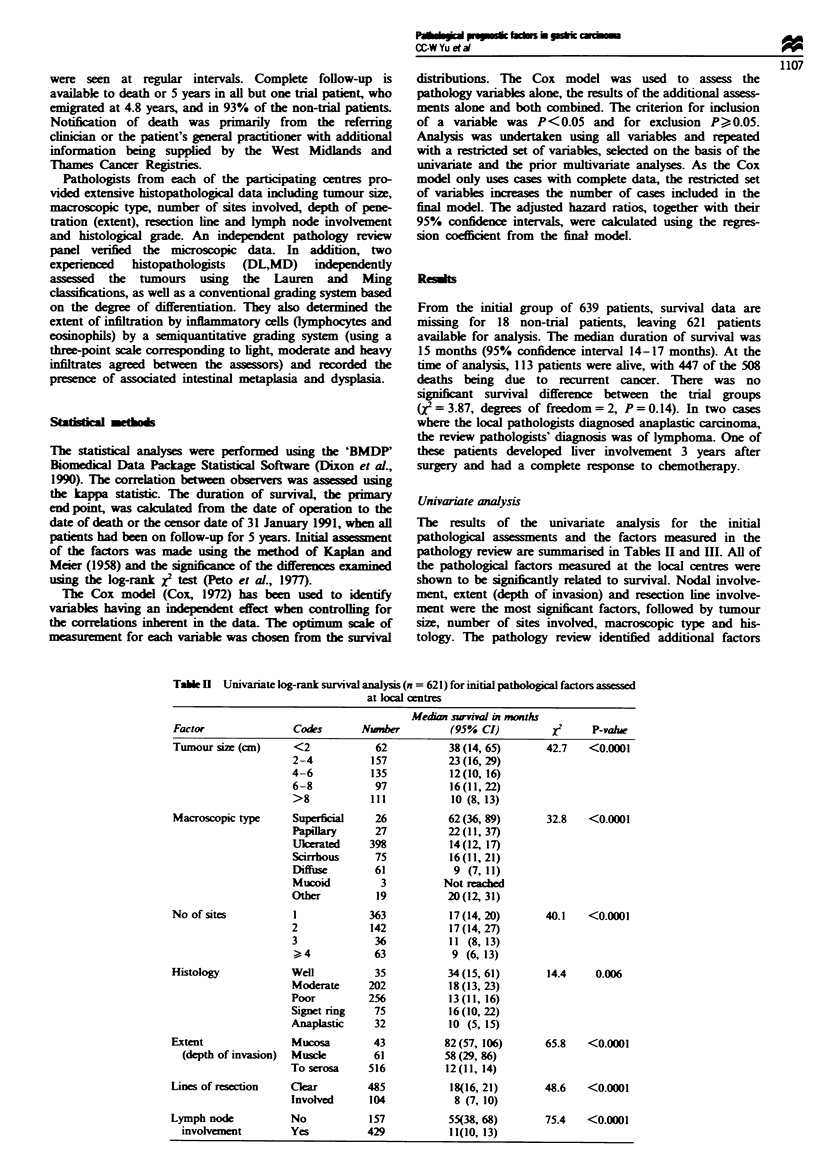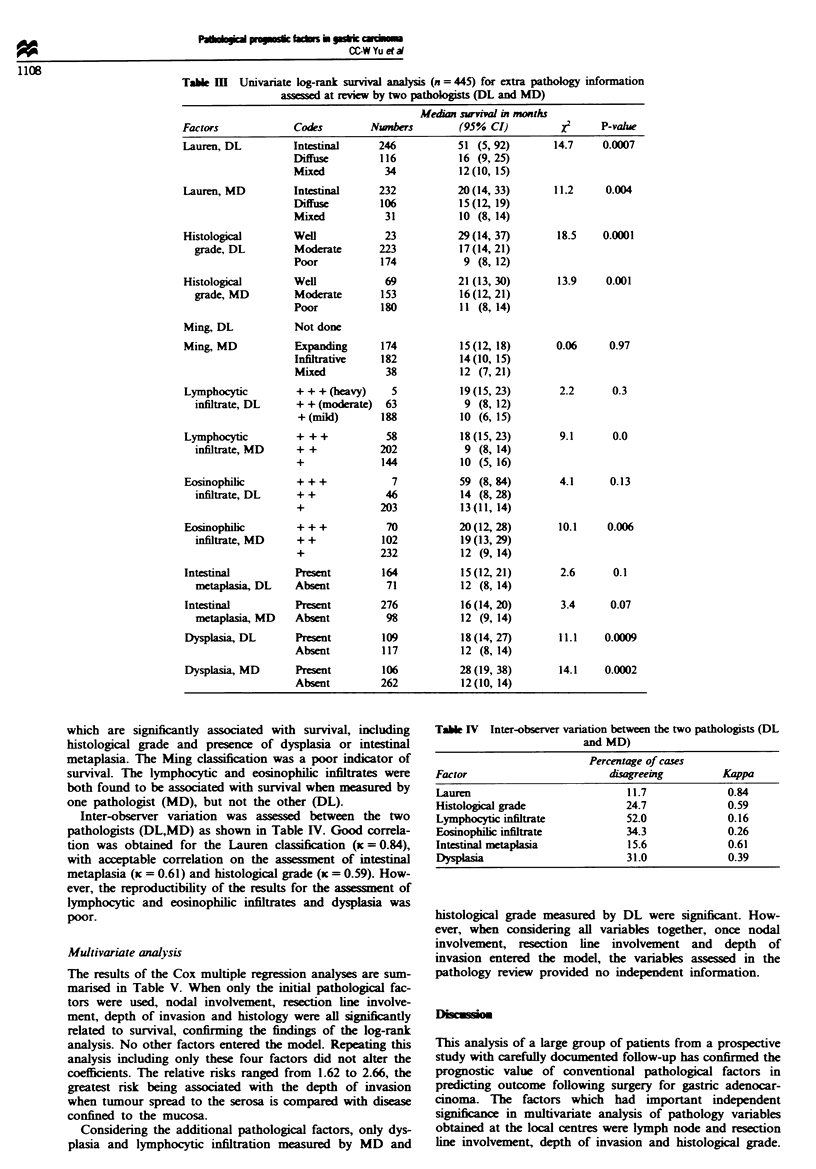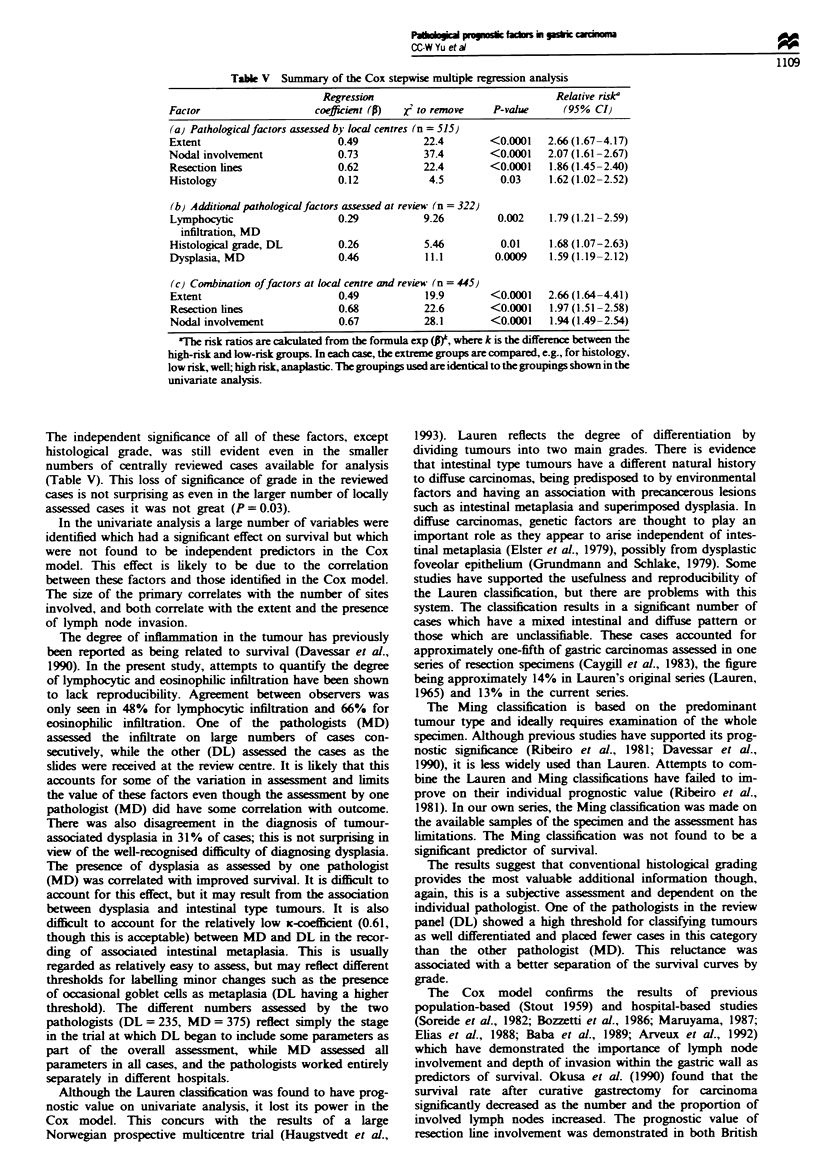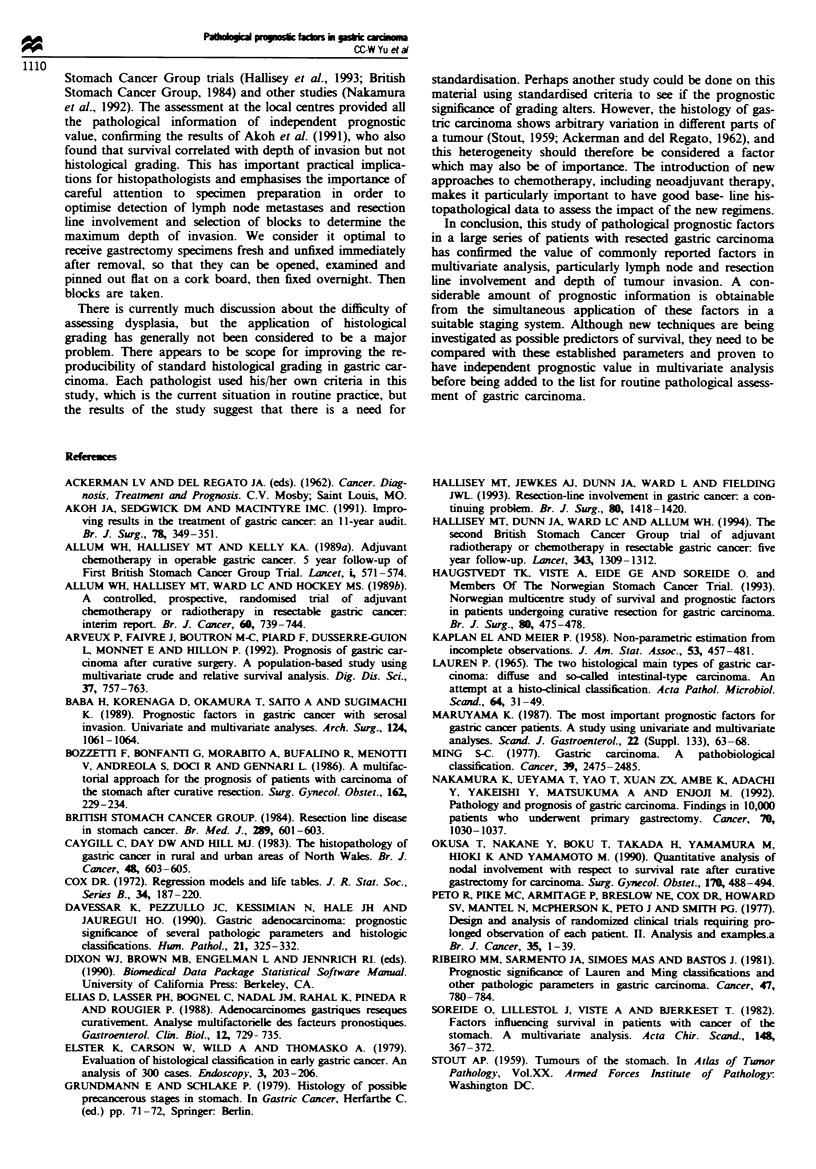# Pathological prognostic factors in the second British Stomach Cancer Group trial of adjuvant therapy in resectable gastric cancer.

**DOI:** 10.1038/bjc.1995.214

**Published:** 1995-05

**Authors:** C. C. Yu, D. A. Levison, J. A. Dunn, L. C. Ward, M. Demonakou, W. H. Allum, M. T. Hallisey

**Affiliations:** Department of Histopathology, UMDS, London, UK.

## Abstract

The second British Stomach Cancer Group trial was a prospective randomised controlled trial of adjuvant radiotherapy or cytotoxic chemotherapy after gastrectomy for adenocarcinoma. It recruited between 1981 and 1986. No survival advantage has been demonstrated for the patients receiving either type of adjuvant therapy compared with those undergoing surgery alone. We report on 436 patients randomised into the trial together with 203 patients, who did not fulfil the trial criteria, referred to the trial. A univariate (log-rank) analysis of pathological factors obtained from the local referring centres showed that tumour size, macroscopic type, number os sites involved, depth of invasion, involvement of resection lines and lymph nodes and histological grade were significant determinants of survival. Histological review by two experienced histopathologists found that the Lauren classification and histological grade, but not the Ming classification, were significant prognostic factors. The degree of lymphocytic and eosinophilic infiltration and presence of dysplasia assessed by one of the pathologists showed a significant correlation with survival. However, inter-observer correlation for these histological parameters and grade was poor. Multivariate analysis identified only depth of invasion, resection line and nodal involvement as significant independent pathological variables influencing survival. This study confirms the need for expert preparation of the resected specimen to obtain the important information on depth of invasion and nodal status and also reveals some variation in histological assessment, particularly grading, in gastric carcinoma.


British Journal of Cancer (1995) 71, 1106–1110

© 1995 Stockton Press Ltd All rights reserved 0007–0920/95 $12.00

### Pathological prognostic factors in the second British Stomach Cancer Group trial of adjuvant therapy in resectable gastric cancer

CC-W Yu[1], DA Levison[1], JA Dunn[2], LC Ward[2], M Demonakou[3], WH Allum[4] and MT Hallisey[5]

[1]*Department of Histopathology, UMDS, Guy's Campus London, UK;* [2]*CRC Trials Unit, Queen Elizabeth Hospital, Birmingham, UK;* [3]*Sismanoglion General Hospital, Athens, Greece;* [4]*Department of Surgery, St. Bartholomew's Hospital, London, UK;* [5]*Department of Surgical Oncology, Queen Elizabeth Hospital, Birmingham, UK.*

**Summary** The second British Stomach Cancer Group trial was a prospective randomised controlled trial of adjuvant radiotherapy or cytotoxic chemotherapy after gastrectomy for adenocarcinoma. It recruited between 1981 and 1986. No survival advantage has been demonstrated for the patients receiving either type of adjuvant therapy compared with those undergoing surgery alone. We report on 436 patients randomised into the trial together with 203 patients, who did not fulfil the trial criteria, referred to the trial. A univariate (log-rank) analysis of pathological factors obtained from the local referring centres showed that tumour size, macroscopic type, number of sites involved, depth of invasion, involvement of resection lines and lymph nodes and histological grade were significant determinants of survival. Histological review by two experienced histopathologists found that the Lauren classification and histological grade, but not the Ming classification, were significant prognostic factors. The degree of lymphocytic and eosinophilic infiltration and presence of dysplasia assessed by one of the pathologists showed a significant correlation with survival. However, inter-observer correlation for these histological parameters and grade was poor. Multivariate analysis identified only depth of invasion, resection line and nodal involvement as significant independent pathological variables influencing survival. This study confirms the need for expert preparation of the resected specimen to obtain the important information on depth of invasion and nodal status and also reveals some variation in histological assessment, particularly grading, in gastric carcinoma.

**Keywords:** gastric cancer; pathology; histological classification; multivariate analysis; prognosis

Gastric carcinoma remains a major cause of death within the United Kingdom and, despite the advances in surgical practice, there has been no change in survival over the past 25 years. To try to influence outcome in this disease, the British Stomach Cancer Group has run two trials of adjuvant therapy. The first trial, which recruited from 1976 to 1981, showed no benefit from adjuvant 5-fluorouracil and mitomycin C (Allum *et al.*, 1989*a*). The second trial, which recruited from 1981 to 1986, compared adjuvant chemotherapy using 5-fluorouracil, doxorubicin (Adriamycin) and mitomycin C, or radiotherapy, with surgery alone (Hallisey *et al.*, 1994). In the second trial, various pathological findings were recorded in detail. Histopathologists reporting adenocarcinomas are familiar with grading into three categories: well, moderately and poorly differentiated. However, alternative classification systems are used in some centres. The Lauren classification divides gastric carcinoma into the intestinal and diffuse types (Lauren, 1965). The Ming classification is based on the growth pattern of the tumour: it divides gastric carcinomas into an expanding type and an infiltrative type (Ming, 1977). These grading systems and other pathological parameters have not previously been analysed formally in such a large cohort of patients with detailed follow-up data within the United Kingdom.

### Patients and methods

The organisation of the trial and its results have been described in detail previously (Allum *et al.*, 1989*b*; Hallisey *et al.*, 1994), but these are summarised briefly here. The trial recruited patients aged 15–74 who had a resection for stage II–IVA(i) adenocarcinoma of the stomach from ten centres in the United Kingdom. The staging was undertaken using a trial-specific clinicopathological staging system formulated in 1980 (Table I), and randomisation was based on the surgical and histological assessment of the referring centre. In addition, 123 patients ineligible on criteria other than stage, 68 patients with stage I disease and 12 patients with metastatic disease were also followed up in accordance with the trial protocol. Data were collated and analysis undertaken at the Cancer Research Campaign Trials Unit in Birmingham.

Trial patients were randomised between the three treatment groups: surgery alone, surgery and chemotherapy or surgery plus radiotherapy. There has been no effect of either adjuvant treatment on survival. Wherever possible patients

**Table I** Clinicopathological staging system of gastric adenocarcinoma used in the trial

| *Stage* | *Parameters* |
|---|---|
| I | Mucosa +ve<br>Submucosa +ve or −ve<br>Muscularis propria +ve or −ve<br>**Serosa[a] −ve**<br>**Nodes −ve** |
| II | Mucosa +ve<br>Submucosa +ve<br>Muscularis propria +ve<br>**Serosa[a] +ve**<br>**Nodes −ve** |
| III | Mucosa +ve<br>Submucosa +ve or −ve<br>Muscularis propria +ve or −ve<br>**Serosa[a] +ve or −ve**<br>**Nodes +ve** |
| IVA | Resected |
| (i)[b] | Local residual disease |
| (ii)[b] | Metastatic residual disease |
| IVB | Unresected |

[a]'Serosa' as used here means either subserosal fat involvement or involvement of the serosal surface.
[b]Mostly only determined clinically, some verified histologically.

Correspondence: DA Levison, Department of Pathology, Ninewells Hospital & Medical School, Dundee DD1 9SY, UK
Received 24 June 1994; revised 5 January 1995; accepted 5 January 1995

were seen at regular intervals. Complete follow-up is available to death or 5 years in all but one trial patient, who emigrated at 4.8 years, and in 93% of the non-trial patients. Notification of death was primarily from the referring clinician or the patient's general practitioner with additional information being supplied by the West Midlands and Thames Cancer Registries.

Pathologists from each of the participating centres provided extensive histopathological data including tumour size, macroscopic type, number of sites involved, depth of penetration (extent), resection line and lymph node involvement and histological grade. An independent pathology review panel verified the microscopic data. In addition, two experienced histopathologists (DL,MD) independently assessed the tumours using the Lauren and Ming classifications, as well as a conventional grading system based on the degree of differentiation. They also determined the extent of infiltration by inflammatory cells (lymphocytes and eosinophils) by a semiquantitative grading system (using a three-point scale corresponding to light, moderate and heavy infiltrates agreed between the assessors) and recorded the presence of associated intestinal metaplasia and dysplasia.

### Statistical methods

The statistical analyses were performed using the 'BMDP' Biomedical Data Package Statistical Software (Dixon *et al.*, 1990). The correlation between observers was assessed using the kappa statistic. The duration of survival, the primary end point, was calculated from the date of operation to the date of death or the censor date of 31 January 1991, when all patients had been on follow-up for 5 years. Initial assessment of the factors was made using the method of Kaplan and Meier (1958) and the significance of the differences examined using the log-rank $\chi^2$ test (Peto *et al.*, 1977).

The Cox model (Cox, 1972) has been used to identify variables having an independent effect when controlling for the correlations inherent in the data. The optimum scale of measurement for each variable was chosen from the survival distributions. The Cox model was used to assess the pathology variables alone, the results of the additional assessments alone and both combined. The criterion for inclusion of a variable was $P<0.05$ and for exclusion $P\geqslant0.05$. Analysis was undertaken using all variables and repeated with a restricted set of variables, selected on the basis of the univariate and the prior multivariate analyses. As the Cox model only uses cases with complete data, the restricted set of variables increases the number of cases included in the final model. The adjusted hazard ratios, together with their 95% confidence intervals, were calculated using the regression coefficient from the final model.

### Results

From the initial group of 639 patients, survival data are missing for 18 non-trial patients, leaving 621 patients available for analysis. The median duration of survival was 15 months (95% confidence interval 14–17 months). At the time of analysis, 113 patients were alive, with 447 of the 508 deaths being due to recurrent cancer. There was no significant survival difference between the trial groups ($\chi^2=3.87$, degrees of freedom = 2, $P=0.14$). In two cases where the local pathologists diagnosed anaplastic carcinoma, the review pathologists' diagnosis was of lymphoma. One of these patients developed liver involvement 3 years after surgery and had a complete response to chemotherapy.

### *Univariate analysis*

The results of the univariate analysis for the initial pathological assessments and the factors measured in the pathology review are summarised in Tables II and III. All of the pathological factors measured at the local centres were shown to be significantly related to survival. Nodal involvement, extent (depth of invasion) and resection line involvement were the most significant factors, followed by tumour size, number of sites involved, macroscopic type and histology. The pathology review identified additional factors

**Table II** Univariate log-rank survival analysis ($n$ = 621) for initial pathological factors assessed at local centres

| *Factor* | *Codes* | *Number* | *Median survival in months (95% CI)* | $\chi^2$ | P-*value* |
|---|---|---|---|---|---|
| Tumour size (cm) | <2 | 62 | 38 (14, 65) | 42.7 | <0.0001 |
| | 2–4 | 157 | 23 (16, 29) | | |
| | 4–6 | 135 | 12 (10, 16) | | |
| | 6–8 | 97 | 16 (11, 22) | | |
| | >8 | 111 | 10 (8, 13) | | |
| Macroscopic type | Superficial | 26 | 62 (36, 89) | 32.8 | <0.0001 |
| | Papillary | 27 | 22 (11, 37) | | |
| | Ulcerated | 398 | 14 (12, 17) | | |
| | Scirrhous | 75 | 16 (11, 21) | | |
| | Diffuse | 61 | 9 (7, 11) | | |
| | Mucoid | 3 | Not reached | | |
| | Other | 19 | 20 (12, 31) | | |
| No of sites | 1 | 363 | 17 (14, 20) | 40.1 | <0.0001 |
| | 2 | 142 | 17 (14, 27) | | |
| | 3 | 36 | 11 (8, 13) | | |
| | ≥4 | 63 | 9 (6, 13) | | |
| Histology | Well | 35 | 34 (15, 61) | 14.4 | 0.006 |
| | Moderate | 202 | 18 (13, 23) | | |
| | Poor | 256 | 13 (11, 16) | | |
| | Signet ring | 75 | 16 (10, 22) | | |
| | Anaplastic | 32 | 10 (5, 15) | | |
| Extent (depth of invasion) | Mucosa | 43 | 82 (57, 106) | 65.8 | <0.0001 |
| | Muscle | 61 | 58 (29, 86) | | |
| | To serosa | 516 | 12 (11, 14) | | |
| Lines of resection | Clear | 485 | 18(16, 21) | 48.6 | <0.0001 |
| | Involved | 104 | 8 (7, 10) | | |
| Lymph node involvement | No | 157 | 55(38, 68) | 75.4 | <0.0001 |
| | Yes | 429 | 11(10, 13) | | |

**Table III** Univariate log-rank survival analysis ($n = 445$) for extra pathology information assessed at review by two pathologists (DL and MD)

| *Factors* | *Codes* | *Numbers* | *Median survival in months (95% CI)* | $\chi^2$ | P-*value* |
|---|---|---|---|---|---|
| Lauren, DL | Intestinal | 246 | 51 (5, 92) | 14.7 | 0.0007 |
| | Diffuse | 116 | 16 (9, 25) | | |
| | Mixed | 34 | 12 (10, 15) | | |
| Lauren, MD | Intestinal | 232 | 20 (14, 33) | 11.2 | 0.004 |
| | Diffuse | 106 | 15 (12, 19) | | |
| | Mixed | 31 | 10 (8, 14) | | |
| Histological grade, DL | Well | 23 | 29 (14, 37) | 18.5 | 0.0001 |
| | Moderate | 223 | 17 (14, 21) | | |
| | Poor | 174 | 9 (8, 12) | | |
| Histological grade, MD | Well | 69 | 21 (13, 30) | 13.9 | 0.001 |
| | Moderate | 153 | 16 (12, 21) | | |
| | Poor | 180 | 11 (8, 14) | | |
| Ming, DL | Not done | | | | |
| Ming, MD | Expanding | 174 | 15 (12, 18) | 0.06 | 0.97 |
| | Infiltrative | 182 | 14 (10, 15) | | |
| | Mixed | 38 | 12 (7, 21) | | |
| Lymphocytic infiltrate, DL | + + + (heavy) | 5 | 19 (15, 23) | 2.2 | 0.3 |
| | + + (moderate) | 63 | 9 (8, 12) | | |
| | + (mild) | 188 | 10 (6, 15) | | |
| Lymphocytic infiltrate, MD | + + + | 58 | 18 (15, 23) | 9.1 | 0.0 |
| | + + | 202 | 9 (8, 14) | | |
| | + | 144 | 10 (5, 16) | | |
| Eosinophilic infiltrate, DL | + + + | 7 | 59 (8, 84) | 4.1 | 0.13 |
| | + + | 46 | 14 (8, 28) | | |
| | + | 203 | 13 (11, 14) | | |
| Eosinophilic infiltrate, MD | + + + | 70 | 20 (12, 28) | 10.1 | 0.006 |
| | + + | 102 | 19 (13, 29) | | |
| | + | 232 | 12 (9, 14) | | |
| Intestinal metaplasia, DL | Present | 164 | 15 (12, 21) | 2.6 | 0.1 |
| | Absent | 71 | 12 (8, 14) | | |
| Intestinal metaplasia, MD | Present | 276 | 16 (14, 20) | 3.4 | 0.07 |
| | Absent | 98 | 12 (9, 14) | | |
| Dysplasia, DL | Present | 109 | 18 (14, 27) | 11.1 | 0.0009 |
| | Absent | 117 | 12 (8, 14) | | |
| Dysplasia, MD | Present | 106 | 28 (19, 38) | 14.1 | 0.0002 |
| | Absent | 262 | 12 (10, 14) | | |

which are significantly associated with survival, including histological grade and presence of dysplasia or intestinal metaplasia. The Ming classification was a poor indicator of survival. The lymphocytic and eosinophilic infiltrates were both found to be associated with survival when measured by one pathologist (MD), but not the other (DL).

Inter-observer variation was assessed between the two pathologists (DL,MD) as shown in Table IV. Good correlation was obtained for the Lauren classification ($\kappa = 0.84$), with acceptable correlation on the assessment of intestinal metaplasia ($\kappa = 0.61$) and histological grade ($\kappa = 0.59$). However, the reproductibility of the results for the assessment of lymphocytic and eosinophilic infiltrates and dysplasia was poor.

### *Multivariate analysis*

The results of the Cox multiple regression analyses are summarised in Table V. When only the initial pathological factors were used, nodal involvement, resection line involvement, depth of invasion and histology were all significantly related to survival, confirming the findings of the log-rank analysis. No other factors entered the model. Repeating this analysis including only these four factors did not alter the coefficients. The relative risks ranged from 1.62 to 2.66, the greatest risk being associated with the depth of invasion when tumour spread to the serosa is compared with disease confined to the mucosa.

Considering the additional pathological factors, only dysplasia and lymphocytic infiltration measured by MD and histological grade measured by DL were significant. However, when considering all variables together, once nodal involvement, resection line involvement and depth of invasion entered the model, the variables assessed in the pathology review provided no independent information.

**Table IV** Inter-observer variation between the two pathologists (DL and MD)

| *Factor* | *Percentage of cases disagreeing* | *Kappa* |
|---|---|---|
| Lauren | 11.7 | 0.84 |
| Histological grade | 24.7 | 0.59 |
| Lymphocytic infiltrate | 52.0 | 0.16 |
| Eosinophilic infiltrate | 34.3 | 0.26 |
| Intestinal metaplasia | 15.6 | 0.61 |
| Dysplasia | 31.0 | 0.39 |

### Discussion

This analysis of a large group of patients from a prospective study with carefully documented follow-up has confirmed the prognostic value of conventional pathological factors in predicting outcome following surgery for gastric adenocarcinoma. The factors which had important independent significance in multivariate analysis of pathology variables obtained at the local centres were lymph node and resection line involvement, depth of invasion and histological grade.

Table V Summary of the Cox stepwise multiple regression analysis

| Factor | Regression coefficient (β) | $\chi^2$ to remove | P-value | Relative risk[a] (95% CI) |
|---|---|---|---|---|
| *(a) Pathological factors assessed by local centres (n = 515)* | | | | |
| Extent | 0.49 | 22.4 | <0.0001 | 2.66 (1.67–4.17) |
| Nodal involvement | 0.73 | 37.4 | <0.0001 | 2.07 (1.61–2.67) |
| Resection lines | 0.62 | 22.4 | <0.0001 | 1.86 (1.45–2.40) |
| Histology | 0.12 | 4.5 | 0.03 | 1.62 (1.02–2.52) |
| *(b) Additional pathological factors assessed at review (n = 322)* | | | | |
| Lymphocytic infiltration, MD | 0.29 | 9.26 | 0.002 | 1.79 (1.21–2.59) |
| Histological grade, DL | 0.26 | 5.46 | 0.01 | 1.68 (1.07–2.63) |
| Dysplasia, MD | 0.46 | 11.1 | 0.0009 | 1.59 (1.19–2.12) |
| *(c) Combination of factors at local centre and review (n = 445)* | | | | |
| Extent | 0.49 | 19.9 | <0.0001 | 2.66 (1.64–4.41) |
| Resection lines | 0.68 | 22.6 | <0.0001 | 1.97 (1.51–2.58) |
| Nodal involvement | 0.67 | 28.1 | <0.0001 | 1.94 (1.49–2.54) |

[a]The risk ratios are calculated from the formula exp $(\beta)^k$, where $k$ is the difference between the high-risk and low-risk groups. In each case, the extreme groups are compared, e.g., for histology, low risk, well; high risk, anaplastic. The groupings used are identical to the groupings shown in the univariate analysis.

The independent significance of all of these factors, except histological grade, was still evident even in the smaller numbers of centrally reviewed cases available for analysis (Table V). This loss of significance of grade in the reviewed cases is not surprising as even in the larger number of locally assessed cases it was not great ($P = 0.03$).

In the univariate analysis a large number of variables were identified which had a significant effect on survival but which were not found to be independent predictors in the Cox model. This effect is likely to be due to the correlation between these factors and those identified in the Cox model. The size of the primary correlates with the number of sites involved, and both correlate with the extent and the presence of lymph node invasion.

The degree of inflammation in the tumour has previously been reported as being related to survival (Davessar *et al.*, 1990). In the present study, attempts to quantify the degree of lymphocytic and eosinophilic infiltration have been shown to lack reproducibility. Agreement between observers was only seen in 48% for lymphocytic infiltration and 66% for eosinophilic infiltration. One of the pathologists (MD) assessed the infiltrate on large numbers of cases consecutively, while the other (DL) assessed the cases as the slides were received at the review centre. It is likely that this accounts for some of the variation in assessment and limits the value of these factors even though the assessment by one pathologist (MD) did have some correlation with outcome. There was also disagreement in the diagnosis of tumour-associated dysplasia in 31% of cases; this is not surprising in view of the well-recognised difficulty of diagnosing dysplasia. The presence of dysplasia as assessed by one pathologist (MD) was correlated with improved survival. It is difficult to account for this effect, but it may result from the association between dysplasia and intestinal type tumours. It is also difficult to account for the relatively low κ-coefficient (0.61, though this is acceptable) between MD and DL in the recording of associated intestinal metaplasia. This is usually regarded as relatively easy to assess, but may reflect different thresholds for labelling minor changes such as the presence of occasional goblet cells as metaplasia (DL having a higher threshold). The different numbers assessed by the two pathologists (DL = 235, MD = 375) reflect simply the stage in the trial at which DL began to include some parameters as part of the overall assessment, while MD assessed all parameters in all cases, and the pathologists worked entirely separately in different hospitals.

Although the Lauren classification was found to have prognostic value on univariate analysis, it lost its power in the Cox model. This concurs with the results of a large Norwegian prospective multicentre trial (Haugstvedt *et al.*, 1993). Lauren reflects the degree of differentiation by dividing tumours into two main grades. There is evidence that intestinal type tumours have a different natural history to diffuse carcinomas, being predisposed to by environmental factors and having an association with precancerous lesions such as intestinal metaplasia and superimposed dysplasia. In diffuse carcinomas, genetic factors are thought to play an important role as they appear to arise independent of intestinal metaplasia (Elster *et al.*, 1979), possibly from dysplastic foveolar epithelium (Grundmann and Schlake, 1979). Some studies have supported the usefulness and reproducibility of the Lauren classification, but there are problems with this system. The classification results in a significant number of cases which have a mixed intestinal and diffuse pattern or those which are unclassifiable. These cases accounted for approximately one-fifth of gastric carcinomas assessed in one series of resection specimens (Caygill *et al.*, 1983), the figure being approximately 14% in Lauren's original series (Lauren, 1965) and 13% in the current series.

The Ming classification is based on the predominant tumour type and ideally requires examination of the whole specimen. Although previous studies have supported its prognostic significance (Ribeiro *et al.*, 1981; Davessar *et al.*, 1990), it is less widely used than Lauren. Attempts to combine the Lauren and Ming classifications have failed to improve on their individual prognostic value (Ribeiro *et al.*, 1981). In our own series, the Ming classification was made on the available samples of the specimen and the assessment has limitations. The Ming classification was not found to be a significant predictor of survival.

The results suggest that conventional histological grading provides the most valuable additional information though, again, this is a subjective assessment and dependent on the individual pathologist. One of the pathologists in the review panel (DL) showed a high threshold for classifying tumours as well differentiated and placed fewer cases in this category than the other pathologist (MD). This reluctance was associated with a better separation of the survival curves by grade.

The Cox model confirms the results of previous population-based (Stout 1959) and hospital-based studies (Soreide *et al.*, 1982; Bozzetti *et al.*, 1986; Maruyama, 1987; Elias *et al.*, 1988; Baba *et al.*, 1989; Arveux *et al.*, 1992) which have demonstrated the importance of lymph node involvement and depth of invasion within the gastric wall as predictors of survival. Okusa *et al.* (1990) found that the survival rate after curative gastrectomy for carcinoma significantly decreased as the number and the proportion of involved lymph nodes increased. The prognostic value of resection line involvement was demonstrated in both British

Stomach Cancer Group trials (Hallisey *et al.*, 1993; British Stomach Cancer Group, 1984) and other studies (Nakamura *et al.*, 1992). The assessment at the local centres provided all the pathological information of independent prognostic value, confirming the results of Akoh *et al.* (1991), who also found that survival correlated with depth of invasion but not histological grading. This has important practical implications for histopathologists and emphasises the importance of careful attention to specimen preparation in order to optimise detection of lymph node metastases and resection line involvement and selection of blocks to determine the maximum depth of invasion. We consider it optimal to receive gastrectomy specimens fresh and unfixed immediately after removal, so that they can be opened, examined and pinned out flat on a cork board, then fixed overnight. Then blocks are taken.

There is currently much discussion about the difficulty of assessing dysplasia, but the application of histological grading has generally not been considered to be a major problem. There appears to be scope for improving the reproducibility of standard histological grading in gastric carcinoma. Each pathologist used his/her own criteria in this study, which is the current situation in routine practice, but the results of the study suggest that there is a need for standardisation. Perhaps another study could be done on this material using standardised criteria to see if the prognostic significance of grading alters. However, the histology of gastric carcinoma shows arbitrary variation in different parts of a tumour (Stout, 1959; Ackerman and del Regato, 1962), and this heterogeneity should therefore be considered a factor which may also be of importance. The introduction of new approaches to chemotherapy, including neoadjuvant therapy, makes it particularly important to have good base- line histopathological data to assess the impact of the new regimens.

In conclusion, this study of pathological prognostic factors in a large series of patients with resected gastric carcinoma has confirmed the value of commonly reported factors in multivariate analysis, particularly lymph node and resection line involvement and depth of tumour invasion. A considerable amount of prognostic information is obtainable from the simultaneous application of these factors in a suitable staging system. Although new techniques are being investigated as possible predictors of survival, they need to be compared with these established parameters and proven to have independent prognostic value in multivariate analysis before being added to the list for routine pathological assessment of gastric carcinoma.

### References

ACKERMAN LV AND DEL REGATO JA. (eds). (1962). *Cancer. Diagnosis, Treatment and Prognosis.* C.V. Mosby; Saint Louis, MO.

AKOH JA, SEDGWICK DM AND MACINTYRE IMC. (1991). Improving results in the treatment of gastric cancer: an 11-year audit. *Br. J. Surg.*, **78**, 349–351.

ALLUM WH, HALLISEY MT AND KELLY KA. (1989*a*). Adjuvant chemotherapy in operable gastric cancer. 5 year follow-up of First British Stomach Cancer Group Trial. *Lancet*, **i**, 571–574.

ALLUM WH, HALLISEY MT, WARD LC AND HOCKEY MS. (1989*b*). A controlled, prospective, randomised trial of adjuvant chemotherapy or radiotherapy in resectable gastric cancer: interim report. *Br. J. Cancer*, **60**, 739–744.

ARVEUX P, FAIVRE J, BOUTRON M-C, PIARD F, DUSSERRE-GUION L, MONNET E AND HILLON P. (1992). Prognosis of gastric carcinoma after curative surgery. A population-based study using multivariate crude and relative survival analysis. *Dig. Dis. Sci.*, **37**, 757–763.

BABA H, KORENAGA D, OKAMURA T, SAITO A AND SUGIMACHI K. (1989). Prognostic factors in gastric cancer with serosal invasion. Univariate and multivariate analyses. *Arch. Surg.*, **124**, 1061–1064.

BOZZETTI F, BONFANTI G, MORABITO A, BUFALINO R, MENOTTI V, ANDREOLA S, DOCI R AND GENNARI L. (1986). A multifactorial approach for the prognosis of patients with carcinoma of the stomach after curative resection. *Surg. Gynecol. Obstet.*, **162**, 229–234.

BRITISH STOMACH CANCER GROUP. (1984). Resection line disease in stomach cancer. *Br. Med. J.*, **289**, 601–603.

CAYGILL C, DAY DW AND HILL MJ. (1983). The histopathology of gastric cancer in rural and urban areas of North Wales. *Br. J. Cancer*, **48**, 603–605.

COX DR. (1972). Regression models and life tables. *J. R. Stat. Soc., Series B.*, **34**, 187–220.

DAVESSAR K, PEZZULLO JC, KESSIMIAN N, HALE JH AND JAUREGUI HO. (1990). Gastric adenocarcinoma: prognostic significance of several pathologic parameters and histologic classifications. *Hum. Pathol.*, **21**, 325–332.

DIXON WJ, BROWN MB, ENGELMAN L AND JENNRICH RI. (eds). (1990). *Biomedical Data Package Statistical Software Manual.* University of California Press: Berkeley, CA.

ELIAS D, LASSER PH, BOGNEL C, NADAL JM, RAHAL K, PINEDA R AND ROUGIER P. (1988). Adenocarcinomes gastriques reseques curativement. Analyse multifactorielle des facteurs pronostiques. *Gastroenterol. Clin. Biol.*, **12**, 729-735.

ELSTER K, CARSON W, WILD A AND THOMASKO A. (1979). Evaluation of histological classification in early gastric cancer. An analysis of 300 cases. *Endoscopy*, **3**, 203–206.

GRUNDMANN E AND SCHLAKE P. (1979). Histology of possible precancerous stages in stomach. In *Gastric Cancer*, Herfarthe C. (ed.) pp. 71–72, Springer: Berlin.

HALLISEY MT, JEWKES AJ, DUNN JA, WARD L AND FIELDING JWL. (1993). Resection-line involvement in gastric cancer: a continuing problem. *Br. J. Surg.*, **80**, 1418–1420.

HALLISEY MT, DUNN JA, WARD LC AND ALLUM WH. (1994). The second British Stomach Cancer Group trial of adjuvant radiotherapy or chemotherapy in resectable gastric cancer: five year follow-up. *Lancet*, **343**, 1309–1312.

HAUGSTVEDT TK, VISTE A, EIDE GE AND SOREIDE O. and Members Of The Norwegian Stomach Cancer Trial. (1993). Norwegian multicentre study of survival and prognostic factors in patients undergoing curative resection for gastric carcinoma. *Br. J. Surg.*, **80**, 475–478.

KAPLAN EL AND MEIER P. (1958). Non-parametric estimation from incomplete observations. *J. Am. Stat. Assoc.*, **53**, 457–481.

LAUREN P. (1965). The two histological main types of gastric carcinoma: diffuse and so-called intestinal-type carcinoma. An attempt at a histo-clinical classification. *Acta Pathol. Microbiol. Scand.*, **64**, 31–49.

MARUYAMA K. (1987). The most important prognostic factors for gastric cancer patients. A study using univariate and multivariate analyses. *Scand. J. Gastroenterol.*, **22** (Suppl. 133), 63–68.

MING S-C. (1977). Gastric carcinoma. A pathobiological classification. *Cancer*, **39**, 2475–2485.

NAKAMURA K, UEYAMA T, YAO T, XUAN ZX, AMBE K, ADACHI Y, YAKEISHI Y, MATSUKUMA A AND ENJOJI M. (1992). Pathology and prognosis of gastric carcinoma. Findings in 10,000 patients who underwent primary gastrectomy. *Cancer*, **70**, 1030–1037.

OKUSA T, NAKANE Y, BOKU T, TAKADA H, YAMAMURA M, HIOKI K AND YAMAMOTO M. (1990). Quantitative analysis of nodal involvement with respect to survival rate after curative gastrectomy for carcinoma. *Surg. Gynecol. Obstet.*, **170**, 488–494.

PETO R, PIKE MC, ARMITAGE P, BRESLOW NE, COX DR, HOWARD SV, MANTEL N, McPHERSON K, PETO J AND SMITH PG. (1977). Design and analysis of randomized clinical trials requiring prolonged observation of each patient. II. Analysis and examples.a *Br. J. Cancer*, **35**, 1–39.

RIBEIRO MM, SARMENTO JA, SIMOES MAS AND BASTOS J. (1981). Prognostic significance of Lauren and Ming classifications and other pathologic parameters in gastric carcinoma. *Cancer*, **47**, 780–784.

SOREIDE O, LILLESTOL J, VISTE A AND BJERKESET T. (1982). Factors influencing survival in patients with cancer of the stomach. A multivariate analysis. *Acta Chir. Scand.*, **148**, 367–372.

STOUT AP. (1959). Tumours of the stomach. In *Atlas of Tumor Pathology*, Vol.XX. *Armed Forces Institute of Pathology*: Washington DC.